# Progress in Surgery

**Published:** 1895-03-30

**Authors:** 


### PROGRESS IN SURGERY.

### GENITO-URINARY SURGERY.

**Hypertrophy of the Prostate.**—The treatment of enlargement of the prostate by castration has engaged considerable attention in all countries, and seems now established on a satisfactory basis. The idea of treating hypertrophy of the prostate in this way seems to have occurred to White, of Pennsylvania; to Ramm, of Christiania; and to Mansell Moullin in this country, about the same time, as is so often the case with a new method of treatment. Certainly, White, of Pennsylvania, started experiments on dogs, or, rather, arranged for such experiments to be made in October, 1892,[1] and had mentioned the idea to another medical man some months earlier; Moullin suggested it to a patient in November of that year,[2] and Ramm's operations were done in April, 1893.[3] Thus, although White was the first to start experiments, and was the first to call the attention of the profession to the subject, Ramm did the first castrations for hypertrophy of the prostate on the Continent, independently of White's suggestion; and Haynes of Los Angeles, California, in America, at the suggestion of White.

The experiments which White made were performed on dogs. After castration the prostate began to atrophy, first the glandular, and then the muscular elements.[4] It is known that castration in early life prevents the development of the prostate, and later in life causes its atrophy. Moreover, if one testicle is removed one side of the prostate atrophies. In moles, hedgehogs, and other animals, the prostate enlarges during the rutting season. All these facts show that the prostate is a sexual organ, and not in any way part of the urinary apparatus, and that the removal of the chief organ of the sexual system causes atrophy of the secondary ones. Although White had collected these facts, and discussed their bearing on the treatment of prostatic hypertrophy by castration, so cautious was he, that he was for some time undecided as to whether the operation was really justifiable. However, in December, 1893, he saw, in consultation with Dr. F. Fremont Smith, at St. Augustin's, Florida, an apparently hopeless case, with marked cystitis and commencing uræmia, and advised the operation. Fifteen weeks after the operation the patient was cured. He "urinated freely and normally," and there was no cystitis.[5] In the following month White operated himself for the first time. The patient was a medical man, 69 years of age, with a prostate the size of an orange, "who had passed no urine except by catheter for years, and whose urine was loaded with mucus, was offensive, and at short intervals was filled with blood." When the case was reported,[6] fourteen weeks after the operation, he had not begun to pass water without the catheter, but the prostate had returned to its normal size, the lengthening of the urethra had ceased to exist, and the passage of the catheter had become easy and painless, whereas before it was difficult and very painful. Other operators have recorded better results than these, for not only has the enlargement of the organ subsided, but the voluntary power of micturition has returned.

Twelve cases have now been operated on with marked success.[7] Powel also records a case in which the enlarged prostate atrophied with great improvement in micturition, after castration for the removal of a growth in one testicle, the other being small and apparently useless.

It is a very curious fact that some years ago Mr. Reginald Harrison was asked by a patient to perform castration for hypertrophy of the prostate, but he would not consent,[9] though, as a compromise, he divided the vasa deferentia. The patient stated that benefit immediately followed, but he was lost sight of, and what the further progress of the case was Mr. Harrison does not know. But Haynes, of California, who has been already referred to as having performed the first castration for prostatic hypertrophy in America, divided the vasa deferentia without "definite results."[10] With regard to the effect of division of the vasa deferentia on the growth of the prostate, Dr. Joseph Griffiths writes,[11] "I have found that ligation or division of the vas deferens in dogs neither retards the growth of the testes in the young nor usually interferes with the maintenance of the spermatozoa-producing powers in the full grown."

It is, perhaps, only necessary to refer to two other cases in which castration has been performed. Moullin's case[12] is almost startling in its success. A man 81 was admitted into the London Hospital with retention from enlarged prostate. The gland was enormously enlarged, and no catheter could be passed, the growth blocking the urethra completely. Suprapubic aspiration was performed, and after that the patient passed urine himself, but with great difficulty and very imperfectly, and then cystitis came on. Castration was performed, and Moullin thus describes the effect: "The next day the urine came more freely. Ten days later the prostate was smaller. Three weeks after the operation it had disappeared. An ordinary catheter passed with ease, and without having the handle depressed to any extent, and when the finger was in the rectum all that could be felt was a fusiform thickening extending along the catheter, so small and soft that the catheter could be felt everywhere through it." Swain's case[13] is as remarkable. The disease was very advanced; retention had occurred, and the urine was offensive; but the benefit from the operation was so great as to amount to a practical cure. This case is of especial interest, as an examination of the testes was made, and showed that even at that age they were in full functional activity, as the semen was full of living spermatozoa, and the microscopic structure normal.

In an article on castration for hypertrophy of the prostate, the Editor of the *Univ. of Pennsylvania Press* remarks that "The results have been so uniformly favourable that castration may now be considered a specific for hypertrophy of the prostate. It is necessary, however, to utter a word of caution here. Castration is not indicated in every case of prostatic enlargement or urinary obstruction. To secure uniformly successful results one must be certain that the condition from which the patient is suffering is appropriate for the operation. Cases of prostatic abscess, prostatitis, tumours of the prostate and of the region of the neck of the bladder, and other forms of obstruction in the neighbourhood of the prostate must be distinguished from true prostatic hypertrophy. Without careful discrimination, both the surgeon and the patient will be disappointed, and the operation will unnecessarily be brought into discredit. As it stands to-day, however, in appropriate cases, it appears to mark an advance in the surgery of the prostate, which, when the gravity and the frequency of the condition of hypertrophy are recalled, together with the more or less ineffectual and always dangerous methods of treatment which have prevailed, must be a source of congratulation not only to Professor White but to the profession at large, and to thousands of patients who, having outlived their sexual lives and earned an old age of mental and physical repose and intellectual enjoyment, have had only a few short years of torment and misery to look forward to on account of this hitherto intractable disease."

Some recent advances in the surgical treatment of enlargement of the prostate are described by Mansell Moullin in a paper[14] read before the Eastbourne Medical Society. He considers that the habitual employment of the catheter is often a remedy scarcely less dangerous than the disease. Even if scrupulously clean, it is always liable to set up inflammation of the irritable mucous membrane, and the prostatic pouch is not properly emptied. Moreover the muscular coat is apt to lose its power of contracting after habitual catheterism. Moullin thinks that "habitual catheterism helps in producing the contracted granular kidney that is so often met with in enlargement of the prostate. The persistent irritation of the deep part of the urethra maintains a condition of chronic renal congestion which ultimately ends in this." The great increase in size of the prostatic plexus of veins as age advances is pointed out, and Moullin considers that the congestion is the immediate source of nearly all the symptoms assigned to the enlargement. There are only two methods of operating besides castration which are considered likely to meet with general acceptance—a modification of Bottini's method, devised by Watson of Boston, and McGill's operation, or suprapubic prostatectomy. Bottini's operation consists in the removal of part of the obstruction by means of the galvano cautery, applied by means of an instrument like a sound passed along the urethra, and Watson's modification of it consists in performing the same operation from a perineal incision into the urethra. The operation is occasionally followed by stranguary, and secondary hæmorrhage has occurred. It is not intended for advanced cases, and is really only suitable for a small obstruction, confined to the posterior wall of the urethra at the orifice of the bladder, and Moullin thinks it acts probably more by destruction of the venous plexuses than by actual removal of the growth. He considers McGill's operation more serious, but more thorough, as lateral as well as median lobes can be dealt with equally well, and just the necessary amount removed to open up the urethral entrance. He thinks calculi are much more frequent in cases of prostatic overgrowth than is usually believed, and they also can be removed by this operation. Some operators leave the mucous membrane of the bladder intact, and after opening the bladder above the pubes and inserting the finger as a guide, remove the enlarged prostate by a perineal incision, but do not penetrate from the perineum into the interior bladder. The object is to preserve the wound in the prostate from contamination with septic urine.

The rate of mortality of suprapubic prostatectomy has been very high—20 per cent., but lately Mayo Robson has reported eleven cases with only one death. Bottini has operated upon seventy-seven cases by his method; he cured fifty-two, and improved eleven. Only two died.

The subject of cystostomy in prostatic patients has lately been considered by the Chirurgical Society of Paris. Dr. Tuffier concludes that the operation is urgently needed in acute retention, and should be temporary; and that when chronic cystitis is present, pain, febrile symptoms, or slow progressive intoxication may require it.

The pathology of enlargement of the prostate is fully discussed by Mansell Moullin.[15] The theory that the hypertrophy of this organ is analagous to the growth of uterine fibroids is shown to have no foundation in embryology. The homologue of the uterus is not the prostate, but the prostatic utricle, and, as Mr. Moullin points out, if it is to be found at all, is near the lower ends of the ducts of Gartner. Then, again, Moullin points out that uterine myomata are quite different in structure from enlarged prostates; the former are fibro-myomata, the latter are glandular from the first. The only analogy between them lies in the fact that removal of the ovary in the one case and the testicle in the other will cause a cessation of their growth. Moullin also rejects the theory of Guyon and the French school, that it is merely one of the occurrences in a constitutional disorder which begins as arterial sclerosis, and ends in fibroid degeneration, because it is a glandular not a fibroid change, and is compatible with perfect health, only a few of those whose prostates are enlarged suffering from urinary trouble. Moullin considers that the facts already mentioned in the early part of this paper show that the influence which determines the prostatic overgrowth, whatever it is, comes from the testes, especially as removal of one testis produces corresponding atrophy of one-half of the prostate, he thinks the influence is exerted through the nervous system.

[1] Brit. Med. Journ., Jan. 5, 1895. [2] Ibid, Nov. 5, 1894. [3] Centralblatt für Chirurgie, No. 35 Sept. 2, 1893. [4] Annals of Surgery, Aug. 1 1893, and Brit. Med. Journ., June 23, 1894, p. 1353. [5] Brit. Med. Journ., June 23, 1894, p. 1353. [6] Ibid. [7] Rann's case, loc. cit.; White's case, loc. cit.; Mansell Moullin, Brit. Med. Journ., Nov. 3, 1894; Haynes, loc. cit.; Fremont Smith, loc. cit.; Swain, Brit. Med. Journ., Jan. 5, 1895; Mayer and Haench, Cent. für die Krankheit der Harn und Sexual-Organe, Aug. 23, 1894; Thomas, Pittsburgh Medical Review, Sept., 1894; Ricketts, Cincinnati Lancet, Clinic, Dec. 1, 1894; Lannois, Ann. des Mal. des Org. Gén. Urinaires, Oct., 1894. [8] Brit. Med. Journ., Nov. 18, 1893, p. 1099. [9] Ibid, Sept. 23, 1893, p. 708. [10] Ibid, June 23, 1894, p. 1353. [11] Ibid, Sept. 30, 1893, p. 765. [12] Ibid, Nov. 3, 1894, [13] Ibid, Jan. 5, 1895. [14] Clinical Journal, Dec. 19, 1894. [15] Lancet, Oct. 20, 1894.

